# Tracking a multitude of abilities as they develop

**DOI:** 10.1111/bmsp.12276

**Published:** 2022-06-05

**Authors:** Maria Bolsinova, Matthieu J. S. Brinkhuis, Abe D. Hofman, Gunter Maris

**Affiliations:** ^1^ Department of Methodology and Statistics Tilburg University Tilburg; ^2^ Department of Information and Computing Sciences Utrecht University Utrecht The Netherlands; ^3^ Department of Psychological Methods University of Amsterdam Amsterdam The Netherlands; ^4^ Prowise Budel The Netherlands; ^5^ Tata Consultancy Services Brussels Belgium

## Abstract

Recently, the Urnings algorithm (Bolsinova *et al*.,  2022, *J. R. Stat. Soc. Ser. C Appl. Statistics*, *71*, 91) has been proposed that allows for tracking the development of abilities of the learners and the difficulties of the items in adaptive learning systems. It is a simple and scalable algorithm which is suited for large‐scale applications in which large streams of data are coming into the system and on‐the‐fly updating is needed. Compared to alternatives like the Elo rating system and its extensions, the Urnings rating system allows the uncertainty of the ratings to be evaluated and accounts for adaptive item selection which, if not corrected for, may distort the ratings. In this paper we extend the Urnings algorithm to allow for both between‐item and within‐item multidimensionality. This allows for tracking the development of interrelated abilities both at the individual and the population level. We present formal derivations of the multidimensional Urnings algorithm, illustrate its properties in simulations, and present an application to data from an adaptive learning system for primary school mathematics called Math Garden.

## 1 Introduction

In recent years large‐scale personalized learning has become one of the key ambitions of educational innovation. It is enabled by the development of adaptive learning systems (ALSs) that are designed to dynamically adjust the level or type of practice and instruction material based on an individual learner's ability or skill attainment. Measurement plays an important role in ALSs, since monitoring the development of learners' skills is crucial to adapting the learning and practice material to their level. To optimize feedback, instructions, and suggested learning material, one needs to have accurate and reliable information about what the learners do and do not know. However, the adaptive, dynamic, and large‐scale nature of ALSs poses challenges for traditional measurement models and statistical algorithms, which were not designed to be used in such contexts.

While traditional measurement models have been extended to allow for dynamic change in ability (e.g., Embretson, 1991; Wang, Berger, & Burdick, 2013), the resulting models are increasingly complex, with a growing number of parameters, such that updating them may be not feasible in real time when large streams of data are coming into the system. Therefore, alternative lightweight algorithms are needed for dynamically updating learners' multiple ability levels on‐the‐fly. Furthermore, not only learners' abilities but also the characteristics of the items need to be tracked over time for the purposes of quality control and for tracking whether the relative item difficulty changes over time (i.e., item parameter drift; Glas, 2000). We note that this context, also termed computerized adaptive practice (see, for example, Klinkenberg, Straatemeier, & van der Maas, 2011), is geared towards learning and is quite different from computerized adaptive testing (CAT) with a focus on assessment (e.g., Wainer, 2000), which requires a pre‐calibrated item bank and stable item parameters. However, see Veldkamp, Matteucci, and Eggen (2011) for an approach to using CAT in learning.

There have been relevant recent developments in the field of intelligent tutoring systems, where a variety of learning models have been constructed that both track ability and model how learning progresses (e.g., Cen, Koedinger, & Junker, 2006; Corbett & Anderson, 1994; Pavlik Jr, Cen, & Koedinger, 2009). While promising, a downside of these models is that they have to make assumptions about *how* learning progresses, which in turn may affect the ability of the system to accurately track ability if the chosen model is misspecified. The required inclusion of a specific learning model may be a desirable feature when the learners' developmental paths are well understood, but can be considered problematic in cases where this knowledge is not available and there is a notable risk of misspecifying the shape of the learning trajectories. It is, therefore, desirable and important to have statistical tools that allow practitioners to accurately track the development of abilities over time without making assumptions about how these abilities develop (trackers in this context are defined in Brinkhuis & Maris, 2020). This has the benefit of separating the question of what ability levels respondents have (i.e., tracking ability) from the question of how these abilities develop (i.e., modelling learning). With accurate tracking procedures in place, one can carefully consider different relevant models for describing the observed learning progressions in the system, for example by considering whether all individuals benefit from certain practice material.

A promising recent development in the context of obtaining lightweight algorithms for dynamically tracking ability has been the adaptation of the Elo rating system (Elo, 1978) for educational purposes (Brinkhuis *et al*., 2018; Klinkenberg *et al*., 2011; Pelánek, 2016). Originally developed for competitive chess, Elo is based on a transparent and computationally efficient algorithm. It can be applied to learners practising items in ALSs analogous to players competing each other: If a learner solves an item correctly, then the learner ‘wins’, while if the response is not correct, the item ‘wins’. When viewed this way, Elo can be used for tracking the progress of learners and the change in the item difficulties. The learner's ability rating (θ) and the item's difficulty rating (δ) are updated as follows:^1^

(1) $$\theta_{\text{updated}}=\theta_{\text{current}}+K\left(X-\frac{\exp\left(\theta_{\text{current}}-\delta_{\text{current}}\right)}{1+\exp\left(\theta_{\text{current}}-\delta_{\text{current}}\right)}\right),$$

(2) $$\delta_{\text{updated}}=\delta_{\text{current}}-K\left(X-\frac{\exp\left(\theta_{\text{current}}-\delta_{\text{current}}\right)}{1+\exp\left(\theta_{\text{current}}-\delta_{\text{current}}\right)}\right),$$

where X is the observed response accuracy (1 for correct, 0 for incorrect), the probability of a correct response (i.e., the expected response accuracy) is based on the Rasch model (Rasch, 1960), and *K* is a step‐size factor. The updating does not require any complex computations, which makes the system highly scalable (i.e., very large numbers of learners and items can be managed by the system).

Though not originally presented as such, the Elo algorithm generates a Markov chain for every learner and every item; however, it is not known whether this Markov chain has an invariant distribution (Brinkhuis & Maris, 2020). This makes it difficult to study the statistical properties of the ratings and to use them for testing scientific hypotheses. Moreover, the reliability of the ratings is unknown. Alternatives to Elo have been proposed that allow for some measure of uncertainty of the ratings, such as Glicko (Glickman, 2001) and TrueSkill (Herbrich, Minka, & Graepel, 2006). In these systems, however, the measures of uncertainty are based on approximations and do not use invariant distributions. Another issue with these rating systems is that the adaptive item selection potentially influences the invariant distribution and has to be corrected for, as described by Hofman *et al*. (2020, p. 13), which to our knowledge has not been implemented in the rating systems presented above.

Recently, the Urnings rating system has been proposed (Bolsinova *et al*., 2022) as an alternative to Elo, maintaining the desirable practical properties (simplicity and scalability) of the latter while addressing its undesirable statistical properties (unknown reliability and effect of adaptive selection). The Urnings algorithm uses the same model for the probability correct as in Elo, but the ratings are updated differently. Determining the probabilities of correct responses is conceptualized as an urn problem, where a rating is represented by a number of coloured balls in an urn. After each response the ratings are updated in such a way that their invariant distribution is binomial with the urn size as the number of trials and the inverse‐logit‐transformed ability (difficulty) as the probability parameter, conditional on the total sum of ratings. An important feature of the Urnings algorithm is that it explicitly corrects for adaptive item selection.

While the Urnings rating system combines the benefits of Elo with desirable statistical properties, in its current form an important limitation prevents it from being optimally suited for ALSs. The system has currently been developed for dealing with a single ability dimension, and hence is tied to unidimensional applications. ALSs generally consider a wide range of abilities with both between‐ and within‐item multidimensionality. Hence, developing a multidimensional extension of the Urnings algorithm is of importance for improving its feasibility and practical usefulness of working with ALSs.

In this paper, we propose a multidimensional Urnings rating system. Furthermore, we propose a modification of the algorithm with a more intuitive updating rule which also allows for a principled way of accessing model fit. We will present analytical derivations of the system, show simulation results showcasing its properties, and present an application to the data of an ALS.

## 2 Methods

### 2.1 Unidimensional Urnings algorithm

We first briefly describe the original Urnings algorithm proposed by Bolsinova *et al*. (2022). Under the Rasch model, the response of learner i to item j can be conceptualized as an outcome of the following process: one ball is sampled from an infinite urn of learner i with the proportion of green balls equal to $\pi_{i}=\exp\left(\theta_{i}\right)/\left(1+\exp\left(\theta_{i}\right)\right)$ (with others being red) and another ball is sampled from an infinite urn of item j with the proportion of green balls equal to $\pi_{j}=\exp\left(\delta_{j}\right)/\left(1+\exp\left(\delta_{j}\right)\right)$ until the balls are of different colour; the colour of the ball from the learner's urn determines the outcome: green for correct, red for incorrect. We can express this algorithmically as follows:

*repeat*

$$Y_{i}∼\text{Bernoulli}\left(\pi_{i}\right)$$

$$Y_{j}∼\text{Bernoulli}\left(\pi_{j}\right)$$

*until* $Y_{i}\neq Y_{j}$

*return* $X_{\mathrm{ij}}=Y_{i}=1-Y_{j}$

For this process the probability of a correct response is

(3) $$\mathrm{Pr}\left(X_{\mathrm{ij}}=1\right)=\frac{\pi_{i}\left(1-\pi_{j}\right)}{\pi_{i}\left(1-\pi_{j}\right)+\pi_{j}\left(1-\pi_{i}\right)}=\frac{\exp\left(\theta_{i}-\delta_{j}\right)}{1+\exp\left(\theta_{i}-\delta_{j}\right)}.$$

To track the abilities and difficulties, the modelled process is mimicked by a process based on *tracking urns* of finite size. The configurations of these tracking urns are used to monitor the development of abilities (difficulties). To track the (inverse‐logit‐transformed) ability of learner i (difficulty of item j), the number of green balls in their tracking urn (with others being red) is used, which is denoted by $R_{i}$ ($R_{j}$) and referred to as the ‘Urning’. The urn size, denoted by $n_{i}$ ($n_{j}$), plays the role of a tuning parameter responsible for the bias–variance trade‐off in the urnings, similar to the step‐size factor K in Elo. While extensions of the algorithm with urn sizes changing throughout the activity in the system can be developed, currently the urn sizes need to be specified at the start for each learner (item) and stay stable, but might vary across learners and items. The choice of the urn size can be guided by the desired precision of the urnings (the higher it is, the higher n should be), expected level of activity in the system (the higher it is, the higher n can be) and the expected rate of change in the parameters (the higher it is, the smaller n should be).

The urnings are updated after each observation such that their invariant distribution is a product of binomials with parameters $n_{i}$ ($n_{j}$) and $\pi_{i}$ ($\pi_{j}$), conditional on their total sum, when there is no change in the true values. That is, unlike the Elo ratings for which the invariant distribution is not known, the statistical properties of the urnings are known. When items are selected randomly,^2^ the Urnings algorithm is as follows. With replacement, sample one ball from the learner's tracking urn and one ball from the item's tracking urn until their colour is different. Once the condition is met, replace the sampled balls with the balls matching the observed response with acceptance probability equal to

(4) $$\min\left(1,\frac{R_{i}\left(n_{j}-R_{j}\right)+\left(n_{i}-R_{i}\right)R_{j}}{R_{i}^{*}\left(n_{j}-R_{j}^{*}\right)+\left(n_{i}-R_{i}^{*}\right)R_{j}^{*}}\right),$$

where $R_{i}$ and $R_{j}$ are the current urnings, and $R_{i}^{*}$ and $R_{j}^{*}$ are the proposed values (see Bolsinova *et al*., 2022, for more details).^3^

### 2.2 Multidimensional Urnings algorithm

Extending the Urnings algorithm to measure multiple dimensions requires the *a priori* specification of the structure of the relationship between the items and the abilities (i.e., which items relate to which dimensions and what the non‐zero weights are equal to). Similarly to the extension of the Urnings algorithm for a unidimensional model with unequal weights (Deonovic, Bolsinova, Bechger, & Maris, 2020), here we consider weights that are positive integers. In a compensatory multidimensional item response theory model the probability of a correct response is the following:

(5) $$\mathrm{Pr}\left(X_{\mathrm{ij}}=1\right)=\frac{\exp\left(\sum_{m=1}^{M}w_{\mathrm{jm}}\left(\theta_{\mathrm{im}}-\delta_{j}\right)\right)}{1+\exp\left(\sum_{m=1}^{M}w_{\mathrm{jm}}\left(\theta_{\mathrm{im}}-\delta_{j}\right)\right)},$$

where $w_{\mathrm{jm}}$ is an integer‐valued weight of item j in dimension m, $\theta_{\mathrm{im}}$ is the mth ability of learner i, and M is the number of dimensions.^4^ This model can be viewed as the multidimensional extension of the one‐parameter logistic model (Verhelst & Glas, 1995), where integer‐valued weights are specified in a unidimensional model. The weights quantify the strength of the relationship between the ability and the probability of a correct response. Without any additional prior information one may want to choose same weights (e.g., equal to 1) for dimensions that are expected to be equally important for solving an item, and weights of different values for primary and secondary dimensions for an item (e.g., 2 and 1, respectively).

The model in equation (5) is equivalent to:

(6) $$\mathrm{Pr}\left(X_{\mathrm{ij}}=1\right)=\frac{{\left(1-\pi_{j}\right)}^{W_{j}}\prod_{m}\pi_{\mathrm{im}}^{w_{\mathrm{jm}}}}{{\left(1-\pi_{j}\right)}^{W_{j}}\prod_{k}\pi_{\mathrm{im}}^{w_{\mathrm{jm}}}+\pi_{j}^{W_{j}}\prod_{k}{\left(1-\pi_{\mathrm{im}}\right)}^{w_{\mathrm{jm}}}},$$

where $W_{j}=\sum_{m=1}^{M}w_{\mathrm{jm}}$, and $\pi_{\mathrm{im}}=\exp\left(\theta_{\mathrm{im}}\right)/\left(1+\exp\left(\theta_{\mathrm{im}}\right)\right)$. Here each learner is represented by M urns and the item is represented by a single urn. The conceptualized process behind the response is as follows. Sample $w_{\mathrm{jm}}$ balls from each of the learner's urns and $W_{j}$ balls from the item's urn, until the colours of the $W_{j}$ balls sampled from the learner's urns are the same, yet different from the colours of the $W_{j}$ balls sampled from the item's urn.^5^

Each learner receives multiple tracking urns, while each item receives only one. When a learner responds to an item, the learner's urns for the dimensions with $w_{\mathrm{jm}}\neq 0$ and the item's urn are updated. In addition to allowing for multidimensionality, we propose a slight modification to the basic algorithm such that it does not require the step with acceptance probability as in equation (4). Instead of first sampling balls from the tracking urns and then (potentially) replacing them with the balls matching the observed response, we first add the balls matching the observed response to the tracking urns and then sample balls from them. That is, the algorithm has two steps.

**Step 1.** Add balls matching the observed response to the tracking urns:

(7) $$R_{\mathrm{im}}^{*}=R_{\mathrm{im}}+w_{\mathrm{jm}}X_{\mathrm{ij}},\forall m\in \left[1:M\right],$$

(8) $$R_{j}^{*}=R_{j}+W_{j}\left(1-X_{\mathrm{ij}}\right).$$

**Step 2.** Sample $w_{\mathrm{jm}}$ balls (without replacement) from each learner's urn m and $W_{j}$ balls (without replacement) from the item's urn. If the colours of all the balls sampled from the learner's urns are equal, yet different from the colours of all the balls sampled from the item's urn, remove the sampled balls from the tracking urns. Otherwise return the balls to the urns and repeat sampling until the condition is satisfied. This can be expressed algorithmically as follows:

*repeat*

$$Y_{\mathrm{im}}^{*}∼\text{Hypergeometric}\left(w_{\mathrm{jm}},n_{\mathrm{im}}+w_{\mathrm{jm}},R_{\mathrm{im}}^{*}\right),\forall m\in \left[1:M\right]$$

$$Y_{j}^{*}∼\text{Hypergeometric}\left(W_{j},n_{j}+W_{j},R_{j}^{*}\right)$$

*until* $|\sum_{m}Y_{\mathrm{im}}^{*}-Y_{j}^{*}|=W_{j}$

*return* $\left\{R_{i1}^{**},\ldots ,R_{\mathrm{iM}}^{**},R_{j}^{**}\right\}=\left\{R_{i1}^{*}-Y_{i1}^{*},\ldots ,R_{\mathrm{iM}}^{*}-Y_{\mathrm{iM}}^{*},R_{j}^{*}-Y_{j}^{*}\right\}$where $\left\{R_{i1}^{**},\ldots ,R_{\mathrm{iM}}^{**},R_{j}^{**}\right\}$ are the updated urnings. Note that operationally we do not simulate the whole process in which balls are repeatedly sampled until the condition is satisfied, but simply simulate the outcome of this process (either $\sum_{m}Y_{\mathrm{im}}^{*}=W_{j}$ and $Y_{j}^{*}=0$, or $\sum_{m}Y_{\mathrm{im}}^{*}=0$ and $Y_{j}^{*}=W_{j}$) using the following probability derived from the sampling process:

$$\mathrm{Pr}\left(\sum_{m}Y_{\mathrm{im}}^{*}=W_{j},Y_{j}^{*}=0\;¦\;R_{i}^{*},R_{j}^{*}\right)$$

(9) $$\begin{array}{l} \mathrm{Pr}\left(\sum_{m}Y_{\mathrm{im}}^{*}=W_{j},Y_{j}^{*}=0\;¦\;R_{i}^{*},R_{j}^{*}\right)\\ =\frac{\prod_{\nu =1}^{W_{j}}\left(n_{j}-R_{j}^{*}-v\right)\prod_{m=1}^{M}\prod_{\nu =0}^{w_{\mathrm{jm}}-1}\left(R_{\mathrm{im}}^{*}+v\right)}{\prod_{\nu =1}^{W_{j}}\left(n_{j}-R_{j}^{*}+v\right)\prod_{m=1}^{M}\prod_{\nu =0}^{w_{\mathrm{jm}}-1}\left(R_{\mathrm{im}}^{*}-v\right)+\prod_{\nu =0}^{W_{j}-1}\left(R_{j}^{*}-v\right)\;\prod_{m=1}^{M}\prod_{\nu =1}^{w_{\mathrm{jm}}}\left(n_{\mathrm{im}}-R_{\mathrm{im}}^{*}+v\right)}. \end{array}$$

The algorithm ensures that the urnings have known invariant distributions when the abilities and the difficulties are stable and repeated observations are collected. Theorem 1 states that the distribution of updated urnings is equal to the distribution of the current urnings. The proof is provided in Appendix A.

**Theorem 1.** If

(10) $$\mathrm{Pr}\left(R_{i}=r_{i},R_{j}=r_{j}\right)=\frac{\prod_{m}\left(\begin{array}{c} n_{\mathrm{im}}\\ r_{\mathrm{im}} \end{array}\right)\pi_{\mathrm{im}}^{r_{\mathrm{im}}}{\left(1-\pi_{\mathrm{im}}\right)}^{n_{\mathrm{im}}-r_{\mathrm{im}}}\left(\begin{array}{c} n_{j}\\ r_{j} \end{array}\right)\pi_{j}^{r_{j}}{\left(1-\pi_{j}\right)}^{n_{j}-r_{j}}{ℐ}_{\text{condition}}}{Z},$$

where the condition of the indicator function is that, for each m, $r_{\mathrm{im}}+\left(w_{\mathrm{jm}}/W_{j}\right)r_{j}=r_{+m}$, $r_{\mathrm{im}}$ is divisible by $w_{\mathrm{jm}}$, and $r_{j}$ is divisible by $W_{j}$; and Z is the normalizing constant, then

(11) $$\left(R_{i}^{**},R_{j}^{**}\right)∼\left(R_{i},R_{j}\right).$$

Given the chosen value of $r_{+}$, equation (10) gives a unique invariant distribution for the learner repeatedly answering the item, since every state $\left(r_{i},r_{j}\right)$ which conserves $r_{+}$ can be reached from any other state also satisfying this condition in a finite number of steps (see Bolsinova *et al*., 2022, for details on how the invariant distribution depends $r_{+}$).

Now instead of considering one item–learner pair that repeatedly produces responses, let us consider an ALS with many learners repeatedly matched to different items. Here, the joint distribution of all urnings is proportional to the product of (truncated) binomial distributions with the sums $\sum_{i}R_{\mathrm{im}}+\sum_{j}\left(w_{\mathrm{jm}}/W_{j}\right)R_{j}$ being constant for every m. For the items with $W_{j}>1$ the corresponding binomial is truncated since the distribution is non‐zero only for $r_{j}$ divisible by $W_{j}$. The mean and variance of these truncated binomials can be derived analytically and for large $n_{\mathrm{im}}$s and $n_{j}$s they are very close to those of the corresponding binomials. For the learners the distributions are not truncated if there are some items with $w_{\mathrm{jm}}=1$ in every dimension. Since $r_{+}$ is constant, there is a small negative dependence between the urnings, and the variance of the binomial gives an upper bound for the actual variance. For each learner (item) the expected value of $R_{\mathrm{im}}/n_{\mathrm{im}}$ ($R_{j}/n_{j}$) is extremely close to $\pi_{\mathrm{im}}$ ($\pi_{j}$), therefore $R_{\mathrm{im}}/n_{\mathrm{im}}$ ($R_{j}/n_{j}$) can be used as an estimate of $\pi_{\mathrm{im}}$ ($\pi_{j}$). Knowing the invariant distribution of the urnings allows one to quantify the uncertainty of the estimate of $\pi_{\mathrm{im}}$ ($\pi_{j}$) using confidence intervals.

### 2.3 Adaptive item selection

The main feature of ALSs is that the learning materials and practice items are selected for the learners based on what is known about their ability. Typically, the items are selected based on the current ratings of the learner and the items in the system. Bolsinova *et al*. (2022) and Hofman *et al*. (2020) demonstrated that not correcting for the adaptive item selection can have detrimental consequences for the ratings. If the difficulty of selected items is matched to the learner's ability, then the variance of the ratings will artificially increase. This variance inflation means that while the *rankings* of the difficulties and abilities are intact, the *ratings* themselves are affected. As a result, the predicted probabilities of correct responses are biased (probabilities above (below) .5 are overestimated (underestimated)), which decreases the quality of future item selection.

To our knowledge the Urnings algorithm is the only one that incorporates a correction for adaptive item selection.^6^ We apply the same correction here. For every item i it should be known what the probability of being selected for learner j is. Let us denote this probability by $S_{\mathrm{ij}}\left(R_{i},R_{j},R^{\left(j\right)}\right)$, which is a function of the current urnings of the learner and the item and of the urnings of all other items ($R^{\left(j\right)}$).^7^ To correct for adaptivity, the new values $\left\{R_{i}^{**},R_{j}^{**}\right\}$ are accepted with probability

(12) $$\min\left(1,\frac{S_{\mathrm{ij}}\left(R_{i}^{**},R_{j}^{**},R^{\left(j\right)}\right)}{S_{\mathrm{ij}}\left(R_{i},R_{j},R^{\left(j\right)}\right)}\right).$$

That is, if selecting item i becomes more probable, the proposed values are always accepted, while otherwise the current values are sometimes retained (for proof and details, see Bolsinova *et al*., 2022).

### 2.4 Reference point for the urnings

To compare the urnings over time we need to keep a clearly interpretable reference point across time. The total sum of the urnings per dimension is not a very convenient reference point, because abilities change over time, learners leave the system taking their balls with them, and new learners enter the system. Therefore, instead of keeping the total sum constant (Batchelder, Bershad, & Simpson, 1992, pp. 185–186), we propose to keep the sum of urnings constant for something that is relatively stable over time, namely for the item pool. While individual items might become relatively more or less difficult, the item pool as a whole (or a subset of it) can be assumed to be relatively stable and the change in all the learners and in the individual items can be interpreted in relation to this pool.

We propose for each dimension to consider the subset of items that load only on this dimension as the reference subset to ensure that the urnings have a stable reference point. If the urning of an item from the reference subset needs to be updated upwards or downwards, this is done only when a different item from this subset needs an update in the opposite direction, that is, a pairwise update of the item urnings is performed (Brinkhuis, Bakker, & Maris, 2015, p. 335). The learners' urnings are updated directly after the response, while a queue of items from the reference subset needing an upward or downward update is created that are waiting for another item from the reference subset to need the opposite update. When such an update is needed, the urning of one item randomly selected from the queue is updated. In this way the green balls would be redistributed among the urns in the reference subset and their total number would stay constant. The urnings of the items outside the reference subsets can be updated without queuing. With this modification of the algorithm, the distributions of the item urnings in the reference subset are (truncated) binomial with the constraint on their sum, while the distributions of the urnings of the learners and of the other items are not constrained.

### 2.5 Evaluating appropriateness of the item weights

Theorem 2 formulates an important property of the algorithm which can be used to evaluate model fit (see Appendix B for the proof).

**Theorem 2.** If the model for $\mathrm{Pr}\left(X_{\mathrm{ij}}=1\right)$ is correctly specified and items are selected randomly, then for each possible combination of values for $\left\{R_{i1}^{*},\ldots ,R_{\mathrm{iM}}^{*},R_{j}^{*}\right\}$ the observed proportion of correct responses is equal to the proportion of updates in which the balls sampled from the learner's tracking urn were green:

(13) $$\begin{array}{l} \mathrm{Pr}\left(X_{\mathrm{ij}}=1¦R_{i1}^{*}=r_{1},\ldots ,R_{\mathrm{jM}}^{*}=r_{M},R_{j}^{*}=t\right)\\ =\mathrm{Pr}\left(Y_{j}^{*}=0¦R_{i1}^{*}=r_{1},\ldots ,R_{\mathrm{jM}}^{*}=r_{M},R_{j}^{*}=t\right)\\ =\frac{\prod_{\nu =1}^{W_{j}}\left(n_{j}-t-v\right)\prod_{m=1}^{M}\prod_{\nu =0}^{w_{\mathrm{jm}}-1}\left(r_{m}+v\right)}{\prod_{\nu =1}^{W_{j}}\left(n_{j}-t+v\right)\prod_{m=1}^{M}\prod_{\nu =0}^{w_{\mathrm{jm}}-1}\left(r_{m}-v\right)+\prod_{\nu =0}^{W_{j}-1}\left(t-v\right)\prod_{m=1}^{M}\prod_{\nu =1}^{w_{\mathrm{jm}}}\left(n_{\mathrm{im}}-r_{m}+v\right)}. \end{array}$$

When M>1, evaluating the match between the observed and expected proportions for each of the $\left(\left(n_{j}+W_{j}\right)/W_{j}+1\right)\prod_{m}\left(n_{\mathrm{im}}+w_{\mathrm{jm}}+1\right)$ possible combinations of urning values is impractical and difficult to interpret. Therefore, we propose to evaluate the appropriateness of the item weights by considering each dimension separately. For every combination of values $\left\{r_{m},t\right\}$ for $\left\{R_{\mathrm{im}}^{*},R_{j}^{*}\right\}$ we approximate the expected probability $\mathrm{Pr}\left(Y_{j}^{*}=0\;¦\;R_{\mathrm{im}}^{*}=r_{m},R_{j}^{*}=t\right)$ with the proportion of updates with $Y_{j}^{*}=0$ among those with $R_{\mathrm{im}}^{*}=r_{m}$ and $R_{j}^{*}=t$ and compare it with the corresponding observed proportion of correct responses.

### 2.6 Tracking population development

Tracking ability development over time is of interest not only at the individual level, but also for the population as a whole. Here, one can study how average abilities change over time, how variances of abilities change, and how relationships between abilities develop. Assuming a multivariate normal distribution for the abilities in the population (on the logit scale), it is straightforward to estimate the parameters of this distribution by considering the probability of the urnings taking their particular values given the population parameters:

(14) $$\mathrm{Pr}\left(R=r\;¦\;\mu ,\sum \right)=\prod_{i}\int \prod_{m=1}^{M}\left(\begin{array}{c} n_{\mathrm{im}}\\ r_{\mathrm{im}} \end{array}\right)\frac{\exp{\left(\theta_{m}\right)}^{r_{\mathrm{im}}}}{{\left(1+\exp\left(\theta_{m}\right)\right)}^{n_{\mathrm{im}}}}g\left(\theta ¦\mu ,\sum \right)d\theta ,$$

where μ and ∑ are the mean vector and the covariance matrix of the ability distribution. In Appendix C we describe a Bayesian algorithm for estimating these parameters.^8^

### 2.7 Bayesian inference about ability on the individual level

In addition to frequentist inference based on the point estimates and confidence intervals, one can also obtain posterior distributions of ability of each of the learners in the multiple dimensions. Unlike the simple estimate $R_{\mathrm{im}}/n_{\mathrm{im}}$ which is based only on the urning of the learner in the specific dimension m, the posterior distribution in each dimension would be also based on the information about the other dimensions and the population distribution of ability. The joint posterior of ability in all dimensions is

(15) $$f\left(\theta_{i}\;¦\;R_{i1},\ldots ,R_{\mathrm{iM}},\mu ,\sum \right).$$

Note that this distribution is different at every timepoint, since the urnings of the persons differ across time and the mean and the covariance matrix are estimated separately for different timepoints. Given the estimates of the population parameters, one can obtain samples from the posterior distribution in equation (15) by following Step 1 of the algorithm used for estimating the population parameters provided in Appendix C. Using these samples, one can compute the estimates (i.e., posterior means) and create credible intervals that reflect uncertainty about the parameter values after taking the urnings in all dimensions and the population distribution into account.

## 3 Simulation study

To demonstrate the properties of the algorithm we carried out a simulation study consisting of two parts. We first consider a scenario in which the abilities and difficulties *do not change* to demonstrate that the urnings follow their theoretical invariant distribution and to illustrate how the appropriateness of item weights can be evaluated. Then we consider a scenario in which abilities *do change* over time to show how the algorithm tracks ability development at the individual and population level.

### 3.1 Part 1: Unchanging abilities and difficulties

#### 3.1.1 Data generation

An ALS with three dimensions, 5,000 learners and 500 items was simulated. The true values for the abilities on the logit scale were sampled from

$$N_{3}\left(0,\left[\begin{array}{ccc} 1 & 0.5 & 0.5\\ 0.5 & 1 & 0.5\\ 0.5 & 0.5 & 1 \end{array}\right]\right).$$

Item difficulties were set equal to the equally spaced quantiles of $N\left(0,1\right)$. Both between‐item and within‐item multidimensionality was included. Twenty‐five different item types (with 20 items each) were used (see Table 1).

**Table 1:** Item types included in the study: $w_{j1},w_{j2},w_{j3}$ are the weights in the three dimensions

$w_{j1}$	$w_{j2}$	$w_{j3}$
1	0	0
2	0	0
3	0	0
0	1	0
0	2	0
0	3	0
0	0	1
0	0	2
0	0	3
1	1	0

$w_{j1}$	$w_{j2}$	$w_{j3}$
2	1	0
1	2	0
2	2	0
1	0	1
2	0	1
1	0	2
2	0	2
0	1	1
0	2	1
0	1	2

$w_{j1}$	$w_{j2}$	$w_{j3}$
0	2	2
1	1	1
2	1	1
1	2	1
1	1	2

In each of the 10,000 sessions each learner responded to one random item and nine adaptively selected items (with probabilities proportional to the expected variance of the item score).^9^ Random selection was included to check the appropriateness of item weights. For illustration we consider three different items: with correctly specified weights ($w_{j}=\left[1,1,0\right]$); with one of the weights too high ($w_{j}=\left[2,1,0\right]$ instead of $\left[1,1,0\right]$); and with one of the weights too low ($w_{j}=\left[1,1,0\right]$ instead of $\left[2,1,0\right]$).

The urn sizes were set to 20 for the learners and 204 for the items.^10^ The urn size was larger for the items because their urnings are updated more often than those of the learners. For each dimension the items that load only on that dimension were used as the reference subset.

#### 3.1.2 Results

**Figure 1:**
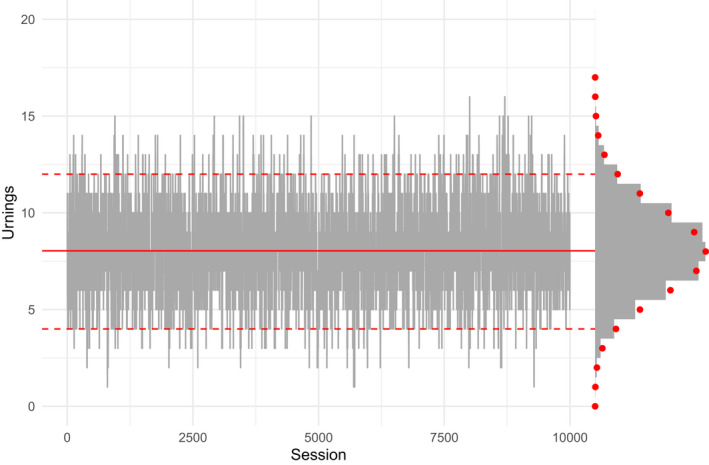
Traceplot of urning values for a single learner in one dimension. The theoretical mean is indicated by a solid red line. The 2.5th and 97.5th percentiles of the binomial distribution are indicated by dashed red lines. The distribution of observed urnings across sessions is displayed by the histogram, overlayed with the theoretical distribution in red dots.

Figure 1 demonstrates for a single $R_{\mathrm{im}}$ that the distribution of the urning is indeed very close to $\text{Binomial}\left(n_{\mathrm{im}},\pi_{\mathrm{im}}\right)$. The last urning values in each of the 10,000 sessions fluctuate around the theoretical mean (solid red line) and about 95% of these values lie within the theoretical bounds (dashed red lines). The observed distribution of the urnings (indicated by the histogram) hardly deviates from the theoretical distribution (indicated by the red dots).

**Figure 2:**
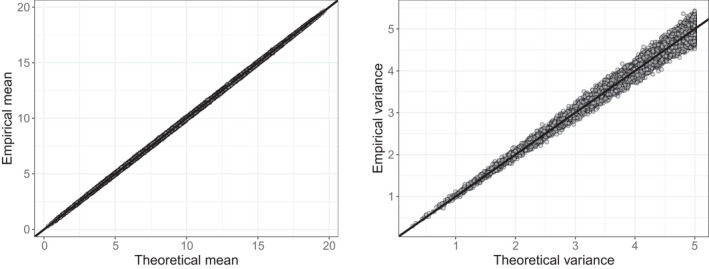
Empirical (y‐axis) and theoretical (x‐axis) means (left) and variances (right) of the urnings of the learners.

**Figure 3:**
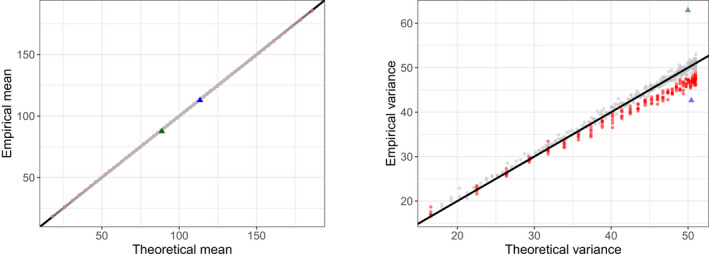
Empirical (y‐axis) and theoretical (x‐axis) means (left) and variances (right) of the urnings of the items. The items indicated in red are the items included in the reference subsets. The items for which the weights were misspecified are indicated in blue (one of the weights is too large) and green (one of the weights is too small) triangles.

Figure 2 shows that for all learners in all dimensions the mean and the variance of the urnings across the sessions are very close to the theoretical values. On the item side (see Figure 3), this is also the case for the items outside of the reference subsets with correctly specified weights (indicated by black dots). As expected, in the reference subsets the variances of the urnings are smaller than those of the (truncated) binomial distributions due to the negative dependence between them (see red dots in Figure 3b). For the items with misspecified weights the means are correctly recovered, but the variances are larger (smaller) than the theoretical variances when $w_{j1}$ is too high (low) (see the blue and green triangles in Figure 3b).

**Figure 4:**
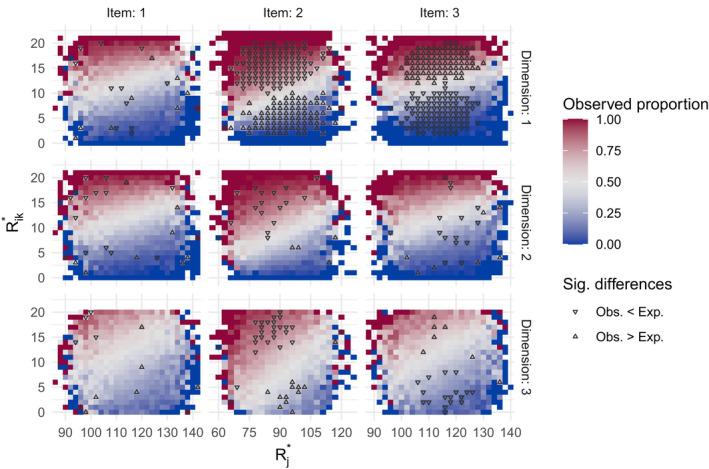
Evaluating appropriateness of item weights. For each of the three items in each of the three dimensions we consider different possible combinations of the urning values for the item and a learner after the first step of the algorithm and compare the observed proportion correct among the responses with such a combination of $R_{j}^{*}$ and $R_{\mathrm{im}}^{*}$ (indicated by different colours) with the expected proportion (Δ (∇) indicates that the observed proportion is significantly larger (smaller) than the expected proportion).

Figure 4 demonstrates how the appropriateness of the weights is checked. The rows and columns represent the three dimensions and the three different items, respectively. For each combination of $R_{j}^{*}$ (x‐axis) and $R_{\mathrm{im}}^{*}$ (y‐axis) the colour represents the observed proportion of correct responses among the responses with such a combination of $R_{j}^{*}$ and $R_{\mathrm{im}}^{*}$ (i.e., urning values after the first step of the algorithm) under random item selection. The combinations for which the expected proportion was significantly smaller (larger) than the observed proportion are indicated by Δ (∇).^11^ For the item with correct weights (first column) there are only a few significant deviations and there is no pattern in them. For the item with $w_{j1}$ too large (second column), for m=1 the deviation is positive and significant for many cells with the observed proportion larger than .5 (∇ in the red cells), and *vice versa* where it is smaller than .5 (Δ in the blue cells). Hence, the strength of the relationship between the ability and $\mathrm{Pr}\left(X_{\mathrm{ij}}=1\right)$ is overestimated. The opposite pattern (i.e., ∇ in the blue cells and Δ in the red cells) is seen for m=1 for the item with $w_{j1}$ too small (third column). Here, the relationship between ability and $\mathrm{Pr}\left(X_{\mathrm{ij}}=1\right)$ is underestimated. Similar but weaker patterns are present for the other dimensions, since all dimensions are correlated and therefore the effect of a misspecification is carried over.

### 3.2 Part 2: Changing abilities

#### 3.2.1 Data generation

This simulation includes three abilities of individuals that change gradually over time, while the population ability distribution is multivariate normal (on the logit scale) at each timepoint. In addition, two specific effects are simulated. First, the variances of ability are simulated to increase over time, creating a so‐called Matthew effect. Second, correlations increase over time to simulate an increasing positive manifold (e.g., Hofman *et al*., 2020; Savi, Marsman, van der Maas, & Maris, 2019). Abilities for 1,000 unique learners at 200 timepoints were generated. Specific details of how the data were generated are provided in Appendix D.

The combination of two factors is important for how well development can be tracked: urn size and how actively the learners use the system. Three levels of activity – low (g=5 items per timepoint), medium (g=15) and high (g=45) – and three urn sizes – small (n=5), medium (n=15) and large (n=45) – were considered. Nine groups of learners (matching the combinations of these factors) with the same underlying abilities were simulated.

From the 500 items 50% had constant difficulty, 25% increased linearly in difficulty by 0.5 on the logit scale from t=0 to t=200, and 25% decreased in difficulty by the same amount. Item difficulties at t=100 were set to be equal to the equally spaced quantiles of $N\left(0,1\right)$. The same types of items as in Part 1 were used, without any weight misspecifications. The average item difficulty in each reference subset was constant and all the change in the individual abilities and difficulties can be interpreted in relation to these constants. The item urn size was set to 204.

#### 3.2.2 Results

**Figure 5:**
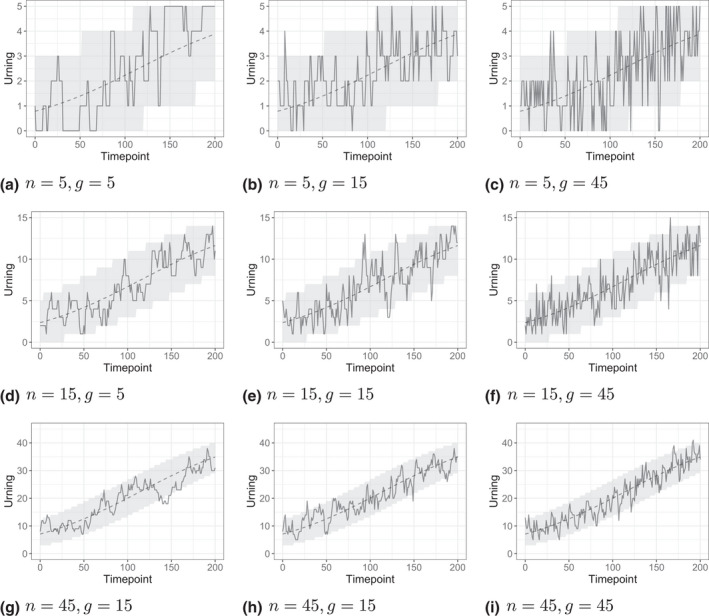
Traceplots of the urnings of nine persons (solid lines) with the same underlying pattern of development of ability (dashed lines) but different level of activity in the ALS (g) and different urn size (n). The dotted lines indicate the expected bounds for the binomial distribution of the urnings.

Figure 5 shows the traceplots of the urnings of nine learners (solid lines) with the same underlying pattern of development of ability ($n/\left(1+\exp\left(-\theta \right)\right)$), dashed lines), but with different levels of activity (g) in the ALS and different urn sizes (n). Generally, when n is higher than g, the tracelines show a lot of autocorrelation and are lagging behind the ability development. At the same time, with higher n there is less noise in the urnings, which is expected based on their theoretical bounds (see grey areas).

**Figure 6:**
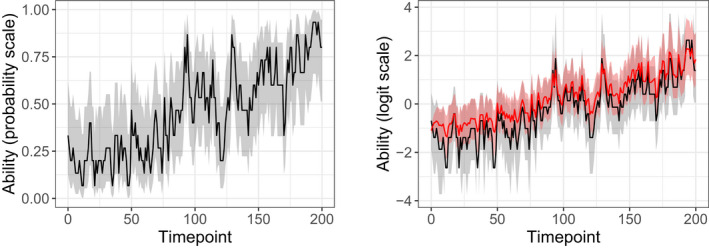
Traceplots for the estimates of ability of one of the learners (with n=15 and g=15) in the first dimension and the associated uncertainty. On the left, the results are shown on the probability scale and are based only on the urnings in the specific dimension (estimate, black line; 95% confidence interval, grey area). On the right, the results are shown on the logit scale and in addition to the results based only on the urnings in the first dimension (black line and grey area), the results based on the urnings in all three dimensions and the population distribution are shown (posterior mean, red line; 95% credible interval, red‐grey area).

Figure 6 illustrates how inferences about the individual ability parameters can be made. For a single person (with n=15 and g=15) it shows the estimates of ability in the first dimension and the uncertainty around them. On the left, the estimates are shown on the probability scale and are based only on the urnings in that dimension. and the uncertainty is given by the 95% confidence intervals.^12^ On the right, the same estimates are shown on the logit scale (black line and grey area) together with the Bayesian estimates (red line) and the corresponding uncertainty quantified with the 95% credible intervals (red‐grey area). Bayesian estimation takes not only the values of $R_{i1}$, but also the urnings in the other two dimensions and the population ability distribution into account, which explains the differences between the two types of estimates and their uncertainty: first, the Bayesian estimates are generally higher because the individual estimates are pooled upwards to the population mean; and second, the Bayesian intervals are less wide because they are based on more information.

Table 2 contains the estimates of the bias and root mean squared error (RMSE) of the individual‐level ability on the probability scale. With the positive development of ability, there is always a negative bias which decreases with g, and is comparable for groups with the same n/g. RMSE, which in addition to bias takes variance into account, mainly depends on urn size (the larger n is, the smaller RMSE is).

**Table 2:** Bias and root mean squared error (RMSE) of the individual abilities (on the probability scale) and of the population parameters (means, standard deviations, and correlations, on the logit scale) for the nine groups of learners with different urn sizes (n) and levels of activity (g)

n	g	Individual ability		Means		*SD*		Correlations	
		Bias	RMSE	Bias	RMSE	Bias	RMSE	Bias	RMSE
5	5	−0.010	0.199	−0.064	0.076	−0.009	0.057	0.003	0.043
	15	−0.005	0.198	−0.034	0.046	−0.026	0.061	0.021	0.050
	45	−0.005	0.198	−0.032	0.048	−0.018	0.060	0.012	0.048
15	5	−0.014	0.116	−0.068	0.070	−0.025	0.037	−0.004	0.021
	15	−0.007	0.115	−0.040	0.045	−0.011	0.028	0.000	0.021
	45	−0.006	0.115	−0.035	0.041	−0.004	0.028	−0.001	0.022
45	5	−0.019	0.070	−0.097	0.099	−0.025	0.030	−0.019	0.023
	15	−0.009	0.067	−0.053	0.055	−0.008	0.016	−0.008	0.015
	45	−0.006	0.066	−0.040	0.043	−0.009	0.017	0.000	0.012

**Figure 7:**
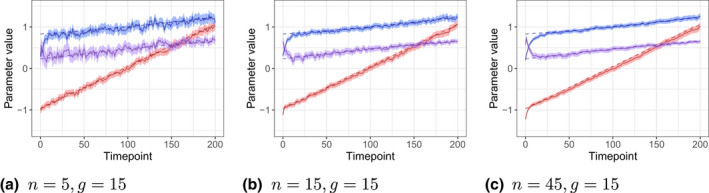
Traceplot for the estimates of the population parameters for ability (red, mean in dimension 1; blue, standard deviation in dimension 1; purple, correlation between dimensions 1 and 2) computed separately for groups of learners with the same level of activity, but different urn sizes. The dashed lines indicate the true development of the population parameters. The coloured areas indicate the 95% credible intervals for the parameters.

Figure 7 shows the development of the population parameters for three of the groups. The algorithm is rather successfully tracking the general development of the parameters. For the mean, the estimates are lagging behind the actual growth, with the severity of the lag increasing with n (keeping g constant). For the standard deviation and the correlation, the lag is visible only for the large urn. Furthermore, with larger n it takes longer to move away from the starting values. The noise in the estimates and the width of the credible intervals decrease with n. Table 2 includes the bias and RMSE of the population‐level estimates (computed starting from t=100 to separate the cold‐start problem from the problem of lagging behind). For the means and standard deviations negative bias is present for all groups, but it increases with n/g. The RMSE follows the pattern of the bias, as the effect of variance decreasing with n is not sufficient to compensate for the increasing bias. For the correlations which do not increase as fast, the bias is close to zero for all conditions, and the RMSE mainly depends on n.

### 3.3 Empirical example

The multidimensional urnings algorithm was applied to data from Math Garden, an ALS for K–12 arithmetics (Brinkhuis *et al*., 2018; Hofman *et al*., 2020; Klinkenberg *et al*., 2011), including several games (e.g., Brinkhuis, Cordes, & Hofman, 2020). Data on 5,860 frequent users of the system with at least 100 responses in three different games – Addition, Multiplication and Speedmix – between 1 September 2018 and 31 May 2020 were selected. In Speedmix children get items that require basic operations to solve, just as in Addition and Multiplication, but have 8 instead of 20 s to respond. While in Math Garden addition and multiplication are tracked without taking the addition and multiplication items from Speedmix into account, here we include these items to track the addition and multiplication dimensions. We consider three dimensions (addition, multiplication, and speed) and let the addition and multiplication items from Speedmix load both on the corresponding substantive dimension and the speed dimension (both with $w_{\mathrm{jm}}=1$). The items from the Addition and Multiplication games only had a weight of 1 for the corresponding substantive dimension.

**Figure 8:**
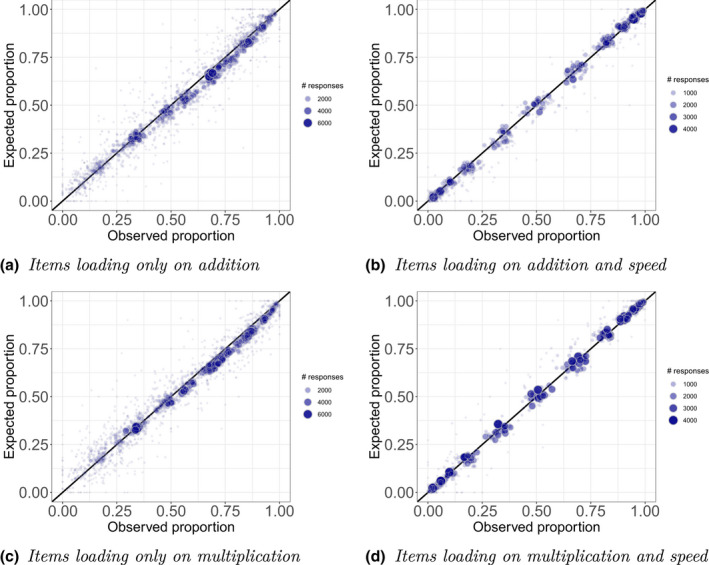
Evaluating model fit for four types of items. Each dot denotes a combination of $R_{j}^{*}$, $R_{i1}^{*}$, $R_{i2}^{*}$ and $R_{i3}^{*}$: among all responses with a particular combination of urning values the observed (x‐axis) and expected (y‐axis) proportion of correct responses are computed.

Figure 8 shows model fit separately for four item types: (a) loading only on addition; (b) loading on addition and speed; (c) loading only on multiplication; and (d) loading on multiplication and speed. Each dot represents a combination of possible values for the item urning and the three learner urnings after the first step of the algorithm (i.e., $R_{j}^{*}$, $R_{i1}^{*}$, $R_{i2}^{*}$ and $R_{i3}^{*}$), and compares the observed and expected proportions of correct responses among all responses with such a combination of the urning values. For all four item types the dots follow the diagonal line. For the items loading only on addition or multiplication, the proportion of correct responses is underestimated where this proportion is relatively high, which could be an indication of the urnings lagging behind the growth of the abilities.

**Figure 9:**
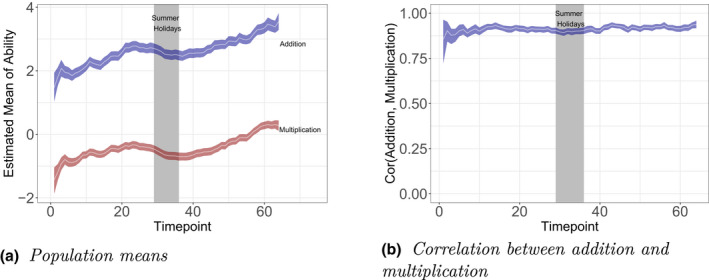
Development of the population parameters over time. The population parameters were estimated at the end of each 10th day in the data set. The white lines indicate the posterior means of the parameters, and the coloured areas indicate the bounds of the 95% credible intervals.

First, we track the ability development at the population level. We focus only on addition and multiplication, because we have a clear reference point only in these dimensions, while the development of the speed dimension over time is not interpretable because of the absence of the reference set of items loading only on this dimension. Figure 9a shows the population means (on the logit scale), while Figure 9b shows the correlation between the dimensions.^13^ The means clearly increase over time, with a dip in the summer holidays. The addition dimension scores higher than the multiplication dimension, which shows that on average the addition items were easier. The correlation between the dimensions was around .90 and stable thought the 2‐year period.

**Figure 10:**
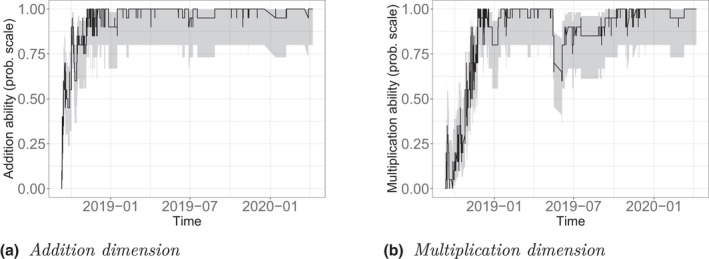
Development of addition and multiplication abilities for a single person.

Second, we track the development of a single learner on both substantive dimensions. Figures 10a,b show the development of the estimates of ability (on the probability scale) in the addition and multiplication dimensions (black lines) and the associated uncertainty quantified by confidence intervals (grey areas). For this person improvement in the addition dimension was faster than in the multiplication dimension.

## 4 Discussion

In this paper we provided a modification and an extension of the recently proposed Urnings algorithm. Given the popularity of multidimensional models, this multidimensional extension of the Urnings algorithm allows for wider applications in ALSs and inference on items not possible before. Earlier approaches avoided within‐item multidimensionality by implementing multiple unidimensional constructs, possibly reducing the ecological validity of such applications in that constructs are practised and tested separately. Using this multidimensional model, more realistic items covering multiple constructs can be offered and modelled.

Another contribution of our paper is that we consider how abilities can be tracked not only at the individual, but also at the population level. Knowing the invariant distributions of the urnings allows us to easily estimate the population parameters (means, standard deviations, and correlations) at any timepoint and evaluate whether they change over time. Importantly, the correlations between the different dimensions are not attenuated due to measurement error, since the uncertainty in the urnings is taken into account when estimating them.

For the development to be interpretable over time in a particular dimension, there needs to be a reference point that is kept constant. In this paper we propose to use the subset of items that load only on the particular dimension as a reference set. If such a set is not available, as was the case for the speed dimension in the empirical example, development of the abilities over time cannot be consistently interpreted. Another important condition for the proper application of the algorithm is correcting for the adaptive item selection. That is, retroactive fitting of the algorithm can only be used for illustrative purposes, as in the empirical example, because some of the results, especially in terms of the development of the population variances, cannot be trusted as the actual development cannot be separated from the potential inflation of the urnings' variance due to not correcting for adaptivity. Thus the algorithm should be built into an ALS such that adaptive selection is based on the urnings and corrected for.

A principled method for evaluating item fit has been developed, a result which can be used more broadly for other fit analyses. For example, by combining data from a specific group rather than the whole population, one can test for differential item functioning. Evaluating person fit is also possible when combining the data on all items for a specific learner.

One of the limitations of the current approach is that item weights need to be specified *a priori* and need to be integer. Though these weight estimates are needed to start, they can be further corrected based on the data. Extending the procedure that we proposed, the appropriateness of the weights can also be monitored continuously to detect any potential changes in the behaviour of the items.

Currently, the sizes of the urns in the model are chosen *a priori*. Smaller urn sizes allow for tracking developments rather quick and coarsely, larger urn sizes allow for more precise measurements yet more responses and little development of ability. Ideally, the algorithm should include a mechanism for changing the urn size based on the change in the behaviour of the learner in the system – for example, to include the frequency of practice, the rate of ability growth and the presence of periods of inactivity as factors for optimizing urn sizes. Such a mechanism is especially of interest in dealing with cold starts of new users, as the influence of the acceptance probability as well as the influence of the paired update procedure are negligible in large‐scale systems. In chess ratings, a pragmatic approach is to start with large step sizes (smaller urns) for beginners, and adapt these later to smaller step sizes (bigger urns). A heuristic working reasonably well in the simulation study is setting the urn size the same as the (expected) number of responses per timepoint, which is similar to the heuristic proposed by Elo (1978, pp. 41–42).

## Conflict of interest

The authors declare no conflict of interest.

## Author contributions

**Maria Bolsinova**: Conceptualization; Data curation; Formal analysis; Funding acquisition; Investigation; Methodology; Software; Project administration; Validation; Writing ‐ original draft; Writing ‐ review and editing. **Matthieu Brinkhuis**: Methodology; Formal analysis; Visualisation; Writing ‐ original draft; Writing ‐ review and editing. **Abe Hofman**: Data curation; Visualisation; Writing ‐ review and editing. **Gunter Maris**: Conceptualisation; Methodology; Writing ‐ review and editing.

## Data availability statement

The data that support the findings of this study are available on request from the corresponding author. The data are not publicly available due to privacy or ethical restrictions.

## Footnotes

^1^
Throughout this manuscript, the term ‘ratings’ is used to indicate either the learner's ability or item's difficulty, and is not used to indicate response choices (e.g., ratings on the Likert scale).

^2^
We will return to the issue of adaptivity in Section 2.3.

^3^
Note, that throughout the iterations the number of green balls ($R_{i}$) or the number of red balls ($n_{i}-R_{i}$) may become zero, but that does not affect the performance of the algorithm. Such border cases would correspond to estimates of $\pi_{i}$ equal to 0 and 1, which are not problematic on the probability scale but would result in improper estimates on the logit scale (−∞ and +∞, respectively), which is one of the reasons why we prefer to work with the probability scale. However, average urnings, which are almost never equal to 0 or $n_{i}$ ($n_{j}$), can be easily transformed to the more common logit scale.

^4^
We use the parametrization with the difference between ability and difficulty inside the parentheses to keep the item parameters of the items that load on multiple dimensions on the same scale as the items that load on a single dimension.

^5^
We note that though this process might seem non‐compensatory, it generates probabilities under the compensatory model in equations (5) and (6).

^6^
An example of a simple tracker that implements a correction by limiting item selection to specific items can be found in Brinkhuis and Maris (2010).

^7^
Note that item selection should be organized in such a way that ergodicity of the Markov chain of the urnings is not effected. That is, all learners and items should be connected to each other, for example if there is a non‐zero probability that person A answers items B and C, and person D answers items B and E, then A, B, C, D and E are connected.

^8^
The probability in equation (14) is proportional to the likelihood of the between‐item multidimensional model with all item difficulties equal to 0 and all discriminations equal to 1. Therefore, any software for estimating multidimensional item response theory models can be used to estimate the population parameters if appropriate constraints on the item parameters are placed. However, using the binomial distributions in estimation would be more efficient.

^9^
When computing the expected probability of a correct response, the number of green and the number of red balls in each urn were increased by 1 to make sure that for none of the item–learner combinations was the probability of a correct response equal to 0 or 1.

^10^
The urn size for the items was chosen such that it is divisible by 1, 2 and 3, which are the possible values for $W_{j}$.

^11^
The significance of the deviation between the observed and expected proportions was tested using the chi‐square test for contingency tables.

^12^
The Wilson score interval (Wilson, 1927) with continuity correction (Newcombe, 1998) was used.

^13^
We do not show the development of the variances because variance inflation may be present due to not correcting for adaptive item selection.

## Appendix A

### Proof of Theorem 1

There are four possible ways in which $\left(R_{i}^{**},R_{j}^{**}\right)$ can be in state $\left(r,t\right)$:

(16)
$$\begin{array}{l} \mathrm{Pr}\left(R_{i}^{**}=r,R_{j}^{**}=t\right)\\ \;=\mathrm{Pr}\left(R_{i}=r,R_{j}=t\right)\mathrm{Pr}\left(X_{\mathrm{ij}}=1\right)\mathrm{Pr}\left(Y_{j}^{*}=0\;¦\;R_{i}^{*}=r+w_{j},R_{j}^{*}=t\right)\\ \;+\mathrm{Pr}\left(R_{i}=r,R_{j}=t\right)\mathrm{Pr}\left(X_{\mathrm{ij}}=0\right)\mathrm{Pr}\left(Y_{j}^{*}=W_{j}\;¦\;R_{i}^{*}=r,R_{j}^{*}=t+W_{j}\right)\\ +\mathrm{Pr}\left(R_{i}^{*}=r+w_{j},R_{j}=t-W_{j}\right)\mathrm{Pr}\left(X_{\mathrm{ij}}=0\right)\mathrm{Pr}\left(Y_{j}^{*}=0\;¦\;R_{i}^{*}=r+w_{j},R_{j}^{*}=t\right)\\ +\mathrm{Pr}\left(R_{i}^{*}=r-w_{j},R_{j}=t+W_{j}\right)\mathrm{Pr}\left(X_{\mathrm{ij}}=1\right)\mathrm{Pr}\left(Y_{j}^{*}=W_{j}\;¦\;R_{i}^{*}=r,R_{j}^{*}=t+W_{j}\right). \end{array}$$

From the process of sampling balls in the second step of the algorithm one can derive that

(17)
$$\mathrm{Pr}\left(Y_{j}^{*}=0¦R_{i}^{*}=r,R_{j}^{*}=t\right)=\frac{\prod_{v=1}^{W_{j}}\left(n_{j}-t-v\right)\prod_{m=1}^{M}\prod_{v=0}^{w_{\mathrm{jm}}-1}\left(r_{m}+v\right)}{\prod_{v=1}^{W_{j}}\left(n_{j}-t+v\right)\prod_{m=1}^{M}\prod_{v=0}^{w_{\mathrm{jm}}-1}\left(r_{m}-v\right)+\prod_{v=0}^{W_{j}-1}\left(t-v\right)\prod_{m=1}^{M}\prod_{v=1}^{w_{\mathrm{jm}}}\left(n_{\mathrm{im}}-r_{m}+v\right)},$$

with an analogous expression for $\mathrm{Pr}\left(Y_{j}^{*}=W_{j}\;¦\;R_{i}^{*}=r,R_{j}^{*}=t+W_{j}\right)$.

Using the binomial identities

(18)
$$\left(\begin{array}{c} n\\ s-w \end{array}\right)=\frac{\prod_{v=0}^{w-1}\left(s-v\right)}{\prod_{v=1}^{w}\left(n-s+v\right)}\left(\begin{array}{c} n\\ s \end{array}\right),$$

(19)
$$\left(\begin{array}{c} n\\ s+w \end{array}\right)=\frac{\prod_{v=0}^{w-1}\left(n-s-v\right)}{\prod_{v=1}^{w}\left(s+v\right)}\left(\begin{array}{c} n\\ s \end{array}\right),$$

together with equations (6) and (17), we can show that

(20)
$$\begin{array}{l} \mathrm{Pr}\left(R_{i}=r+w_{j},R_{j}=t-W_{j}\right)\mathrm{Pr}\left(X_{\mathrm{ij}}=0\right)\mathrm{Pr}\left(Y_{j}^{*}=0\;¦\;R_{i}^{*}=r+w_{j},R_{j}^{*}=t\right)\\ =\mathrm{Pr}\left(R_{i}=r,R_{j}=t\right)\mathrm{Pr}\left(X_{\mathrm{ij}}=1\right)\mathrm{Pr}\left(Y_{j}^{*}=W_{j}\;¦\;R_{i}^{*}=r+w_{j},R_{j}^{*}=t\right), \end{array}$$

from which it follows that the sum of the first and the third element in equation (16) is equal to $\mathrm{Pr}\left(R_{i}=r,R_{j}=t\right)\mathrm{Pr}\left(X_{\mathrm{ij}}=1\right)$. Analogously, it can be shown that the sum of the second and the fourth elements in equation (16) is equal to $\mathrm{Pr}\left(R_{i}=r,R_{j}=t\right)\;\mathrm{Pr}\left(X_{\mathrm{ij}}=0\right)$. Therefore

(21)
$$\begin{array}{r} \mathrm{Pr}\left(R_{i}^{**}=r,R_{j}^{**}=t\right)=\mathrm{Pr}\left(R_{i}=r,R_{j}=t\right)\mathrm{Pr}\left(X_{\mathrm{ij}}=1\right)+\mathrm{Pr}\left(R_{i}=r,R_{j}=t\right)\mathrm{Pr}\left(X_{\mathrm{ij}}=0\right)\\ =\mathrm{Pr}\left(R_{i}=r,R_{j}=t\right), \end{array}$$

which completes the proof.

## Appendix B

### Proof of Theorem 2

There are two possible ways for $\left\{R_{i}^{*},R_{j}^{*}\right\}$ to take values $\left\{r,t\right\}$: from state $\left\{r-w_{j},t\right\}$ with the correct response and from state $\left\{r,t-W_{j}\right\}$ with the incorrect response. Therefore, conditional on $\left\{R_{i}^{*},R_{j}^{*}\right\}$ being equal to $\left\{r,t\right\}$, the probability of a correct response is:

(22)
$$\begin{array}{l} \mathrm{Pr}\left(X_{\mathrm{ij}}=1\;¦\;R_{i}^{*}=r,R_{j}^{*}=t\right)\\ =\frac{\mathrm{Pr}\left(R_{i}=r-w_{j},R_{j}=t\right)\mathrm{Pr}\left(X_{\mathrm{ij}}=1\right)}{\mathrm{Pr}\left(R_{i}=r-w_{j},R_{j}=t\right)\mathrm{Pr}\left(X_{\mathrm{ij}}=1\right)+\mathrm{Pr}\left(R_{i}=r,R_{j}=t-W_{j}\right)\mathrm{Pr}\left(X_{\mathrm{ij}}=0\right)}. \end{array}$$

Using the binomial identity from equation (14) and dividing both the numerator and the denominator by

(23)
$$\frac{\prod_{k}\left(\begin{array}{c} n_{\mathrm{im}}\\ r_{m} \end{array}\right)\pi_{\mathrm{im}}^{r_{m}}{\left(1-\pi_{\mathrm{im}}\right)}^{n+w_{\mathrm{jm}}-r_{m}}\left(\begin{array}{c} n_{j}\\ t \end{array}\right)\pi_{j}^{t}{\left(1-\pi_{j}\right)}^{n_{j}+W_{j}-t}}{\left(\prod_{m}\pi_{\mathrm{im}}^{w_{\mathrm{jm}}}{\left(1-\pi_{j}\right)}^{W_{j}}+\prod_{m}{\left(1-\pi_{\mathrm{im}}\right)}^{w_{\mathrm{im}}}\pi_{j}^{W_{j}}\right)\prod_{v=1}^{W_{j}}\left(n_{j}-t+v\right)\prod_{m=1}^{M}\prod_{v=1}^{w_{\mathrm{jm}}}\left(n_{\mathrm{im}}-r_{m}+v\right)},$$

we obtain the probability

(24)
$$\frac{\prod_{v=1}^{W_{j}}\left(n_{j}-t-v\right)\prod_{m=1}^{M}\prod_{v=0}^{w_{\mathrm{jm}}-1}\left(r_{m}+v\right)}{\prod_{v=1}^{W_{j}}\left(n_{j}-t+v\right)\prod_{m=1}^{M}\prod_{v=0}^{w_{\mathrm{jm}}-1}\left(r_{m}-v\right)+\prod_{v=0}^{W_{j}-1}\left(t-v\right)\;\prod_{m=1}^{M}\prod_{v=1}^{w_{\mathrm{jm}}}\left(n_{\mathrm{im}}-r_{m}+v\right)},$$

which completes the proof.

## Appendix C

### Estimation of population parameters

The population parameters **μ** and ∑ of the distribution of ability (on the logit scale) can be estimated using a Gibbs sampler in which parameters are sampled from their full conditional distributions to obtain samples from the joint posterior $p\left(\mu ,\sum \;¦\;R\right)$. We propose to use an improper prior for **μ**: $p\left(\mu \right)\propto 1$, and a semi‐conjugate low‐informative prior ∑, that is, an inverse‐Wishart distribution with an identity matrix as the scale parameter and the prior degrees of freedom equal to M+2. To simplify the conditional posterior data augmentation is used: we sample from $p\left(\theta ,\mu ,\sum \;¦\;R\right)$ instead of $p\left(\mu ,\sum \;¦\;R\right)$. Below are the steps of the algorithm.

**Step 1.** For each learner i in each dimension m, sample $\theta_{\mathrm{im}}$ from $p\left(\theta_{\mathrm{im}}\;¦\;\theta_{i\left(m\right)},\mu ,\sum ,R_{\mathrm{im}}\right)$, where $\theta_{i\left(m\right)}$ are the abilities of learner i in all dimensions except m. Note that, conditional on $\theta_{i\left(m\right)}$, $\theta_{\mathrm{im}}$ is independent of the urnings in all dimensions other than m. To sample from this distribution the single variable exchange algorithm (Marsman, Maris, Bechger, & Glas, 2017) is used. First sample a candidate value $\theta^{*}$ from the conditional distribution of the multivariate normal $N_{M}\left(\mu ,\sum \right)$ given the values of $\theta_{i\left(m\right)}$, then using this value simulate $R^{*}∼\text{Binomial}\left(n_{\mathrm{im}},1/\left(1+\exp\left(-\theta^{*}\right)\right)\right)$. The probability of accepting $\theta^{*}$ as a new value for $\theta_{\mathrm{im}}$ is $\min\left(1,\exp\left(\left(\theta^{*}-\theta_{\mathrm{im}}\right)\left(R_{\mathrm{im}}-R^{*}\right)\right)\right).$.

**Step 2.** Sample **μ** from $p\left(\mu \;¦\;\theta ,\sum \right)$. Note that, conditional on θ, **μ** is independent of R. This distribution is a multivariate normal with the sample mean vector of θ as the mean vector and $\frac{1}{N}\sum$ as the covariance matrix, where N is the sample size.

**Step 3.** Sample ∑ from $p\left(\sum ¦\;\theta ,\mu \right).$ Note, that conditional on θ, ∑ is independent of R. This distribution is an inverse‐Wishart distribution with the scale parameter equal to $I_{M}+\sum_{i}\left(\theta -\mu \right)\;{\left(\theta -\mu \right)}^{T}$ and N+M+2 degrees of freedom.

## Appendix D

### Details of data generation for Part 2 of the simulation study

To generate a pattern of development with increasing means, standard deviations and correlations, we used a rather simple growth model. First, for each person i, we sampled two latent variables $\left\{\eta_{i},\Delta_{i}\right\}$ from a bivariate normal distribution with mean vector $\left(0,1\right)$, standard deviations equal to $\sqrt{0.5}$ and 0.3, and correlation of .8 (i.e., more able students are learning faster). Second, the ‘general’ ability of each person at timepoint $t\in \left[0:200\right]$ was computed using a linear growth model

$$\eta_{\mathrm{it}}=\eta_{i}+\frac{t-100}{100}\Delta_{i}.$$

That is, the growth model is parameterized in such a way that η is the general ability at timepoint 100, and Δ is the difference in ability between timepoints 0 and 100, and between timepoints 100 and 200. Third, each of the three abilities measured in the learning system were generated: $\theta_{\mathrm{imt}}=\eta_{\mathrm{it}}+\varepsilon_{\mathrm{mi}}$, $\varepsilon_{\mathrm{mi}}∼N\left(0,0.5\right)$, for all $m\in \left[1:3\right]$. For simplicity, the unique component of each ability was generated to be stable across time.
